# Comparative lytic efficacy of rt-PA and ultrasound in porcine versus human clots

**DOI:** 10.1371/journal.pone.0177786

**Published:** 2017-05-17

**Authors:** Shenwen Huang, Himanshu Shekhar, Christy K. Holland

**Affiliations:** 1 Department of Biomedical, Chemical, & Environmental Engineering, College of Engineering and Applied Sciences, University of Cincinnati, Cincinnati, Ohio, United States of America; 2 Division of Cardiovascular Health and Disease, Department of Internal Medicine, University of Cincinnati, Cincinnati, Ohio, United States of America; Monash University, AUSTRALIA

## Abstract

**Introduction:**

Porcine thrombi are employed routinely in preclinical models of ischemic stroke. In this study, we examined the differential lytic susceptibility of porcine and human whole blood clots with and without the use of microbubbles and ultrasound (US) as an adjuvant.

**Materials and methods:**

An *in vitro* system equipped with time-lapse microscopy was used to evaluate recombinant tissue-plasminogen activator (rt-PA) lysis of porcine and human clots in the same species or cross species plasma. Human and porcine whole blood clots were treated with rt-PA and an echo contrast agent, Definity^®^, and exposed to intermittent 120 kHz US.

**Results and conclusions:**

The rt-PA lytic efficacy observed for porcine clots in porcine plasma was 22 times lower than for human clots in human plasma reported previously. Further, porcine clots did not exhibit increased lysis with adjuvant Definity^®^ and US exposure. However, the rt-PA lytic susceptibility of the porcine clots in human plasma was similar to that of human clots in human plasma. Human clots perfused with porcine plasma did not respond to rt-PA, but adjuvant use of Definity^®^ and US enhanced lysis. These results reveal considerable differences in lytic susceptibility of porcine clots and human clots to rt-PA. The use of porcine clot models to test new human thrombolytic therapies may necessitate modulation of coagulation and thrombolytic factors to reflect human hemostasis accurately.

## Introduction

Ischemic stroke affects nearly 700,000 people each year and is the fifth most common cause of death in the United States [1]. Presently, mechanical thrombectomy and intravenous administration of recombinant tissue-type plasminogen activator (rt-PA) are approved by the United States Food and Drug Administration for recanalization of arteries blocked in ischemic stroke. Although these treatments are clinically effective, ischemic stroke patients continue to experience significant morbidity and mortality. Intravenous administration of rt-PA can provide benefit within 3 to 4.5 hours of symptom onset, but treatment exclusion criteria limit administration to only 3.4–5.2% of patients [1–3]. Treated patients display variable outcomes dependent on clot size, site, and composition, with larger, proximal, and more fibrin-rich clots being more resistant to thrombolytic treatment [4–8]. Mechanical thrombectomy, the physical removal of the clot endovascularly, can be effective in treating ischemic stroke for up to 8 hours after symptom onset [9–14]. However, mechanical thrombectomy can only be performed when clots are located in larger cranial vessels. Only an estimated 4–14% of acute ischemic stroke patients are eligible for thrombectomy [15]. Furthermore, many hospitals lack the facilities and expertise to perform this advanced interventional procedure. Patients treated with either modality exhibit similar rates of disability-free recovery (34.8% for intravenous rt-PA *vs* 30.4% for endovascular interventions), as well as similar rates of serious adverse reactions (6% incidence of symptomatic intracerebral hemorrhage [sICH] in both groups) [16]. Some additional benefits have been shown with the initiation of an antiplatelet agent (e.g. aspirin) within 48 hours of symptom onset. Though antiplatelet agents prevent the formation of new thrombi, they do not promote recanalization, and, importantly can increase the incidence of sICH [17,18]. The limitations of the existing treatment modalities motivate continued investigation of alternative strategies to improve efficacy, expand the duration of the treatment window, or increase the size of the eligible patient population.

Sonothrombolysis, the use of ultrasound (US) to promote clot lysis, has been under investigation for enhancing the efficacy of thrombolytic therapy [19–26]. Clinical trials have shown that Doppler US as an adjuvant to rt-PA can moderately increase the likelihood of complete recanalization and functional independence at 3 months [27,28]. Unfortunately, 2-MHz transcranial Doppler US cannot penetrate the skull in about 12–18% of the patient population, due to increased attenuation within the temporal bone [29,30].

Our group has reported enhancement of rt-PA thrombolysis with 120 kHz intermittent ultrasound using a low *in situ* peak-to-peak pressure amplitude (0.44 MPa), which avoids the limitations of temporal bone acoustic window insufficiency [22,31–33]. This approach appears to expedite clot lysis by accelerating penetration of the thrombolytic into the clot and facilitating removal of fibrin degradation products via two distinct mechanical effects: fluid mixing from microbubble activity and vibration from radiation force [22]. Sustained microbubble activity, or stable cavitation, can enhance thrombolysis by increasing the penetration of drug into clots [20,22,34]. Stable cavitation can be nucleated effectively at low acoustic pressures by administration of an US contrast agent (UCA) such as Definity^®^ (Lantheus Medical Imaging, North Billerica, MA, USA). These experiments employed an *in vitro* time-lapse microscopy system [22,23,33] that allowed for careful control of the clot type, surrounding fluid, and US field, as well as precise quantification of thrombolysis and the degree of bubble activity.

Evaluation of US exposure parameters and treatment protocols *in vitro* and in animal models is key to the clinical translation of US as an adjuvant in ischemic stroke therapy. Previously, human and porcine clots have been treated *in vitro* and *ex vivo* [19,20,22,33]. In addition animal models have been developed to test sonothrombolysis [21,24,35] and thrombectomy procedures *in vivo* [36,37]. Highly retracted, dense clots are more resistant to rt-PA lysis and are associated with poor clinical treatment outcomes [38]. Retracted thromboemboli can arise as a result from arterial atheromatous plaques or as a result of cardiac disease (such as atrial fibrillation, patent foramen ovale, cardiac mural thrombi) and migrate to the cerebrovasculature to cause ischemic stroke [39–42]. Our group has reported techniques to develop highly-retracted human and porcine clots for evaluating rt-PA sonothrombolysis *in vitro* [19,22,43].

Although porcine clots are employed routinely in thrombolysis research, the comparative lytic efficacy of rt-PA and intermittent US in porcine versus human clots has not been established. Porcine clots are characterized by a denser fibrin network compared to human clots, and demonstrate higher lytic resistance when treated with human plasmin [44]. Activation of plasminogen to form plasmin relies on the presence of both rt-PA and fibrin [45]. The activation of human and porcine plasminogen by rt-PA in the presence of same species or cross species fibrin, has been compared previously [46]. The resultant porcine plasmin activity was found to be lower than human plasmin activity by almost 10-fold [46]. However, the effect of this differential rt-PA activation on thrombolytic efficacy, with US exposure as an adjuvant, is still unknown.

The objective of this study was to compare thrombolytic efficacy between porcine and human clots exposed to plasma alone, rt-PA, or rt-PA with Definity^®^ exposed to intermittent 120 kHz US as an adjuvant. We also exposed human and porcine clots to either porcine or human plasma to evaluate whether the type of plasminogen affected rt-PA thrombolytic efficacy. An *in vitro* flow model and time-lapse microscopy system was employed to determine lytic efficacy as reported previously [22,23,33].

## Materials and methods

### Preparation of plasma, rt-PA, and ultrasound contrast agents

Human or porcine fresh frozen plasma was pooled prior to experimental use to minimize the effect of individual-to-individual variability. Sodium citrate-anticoagulated fresh frozen pooled porcine plasma was procured from Lampire Biological Laboratories (Pipersville, PA, USA). Porcine plasma was thawed and filtered through 4x4 gauze pads (Fisher Healthcare, Pittsburgh, PA, USA) to remove fibrin clots. Eight units of citrate phosphate double dextrose-anticoagulated fresh frozen human plasma were acquired from the Hoxworth Blood Center (Cincinnati, OH, USA). These units were thawed, pooled, and refrozen as 25 mL aliquots for experimental use. Prior to each experiment, either porcine or human plasma was thawed and warmed to 37°C and allowed to equilibrate to atmospheric gas saturation.

The FDA approved lytic agent rt-PA (Activase, Genentech, San Francisco, CA, USA) was reconstituted with sterile water according to manufacturer instructions to a concentration of 1 mg/mL. One-milliliter aliquots were created and stored at -80°C until use. Aliquots of rt-PA stored according to this protocol have been shown to have stable activity for at least 7 years [47]. Definity^®^ perflutren lipid microspheres were activated according to manufacturer instructions. Specifically, vials of Definity^®^ stored at 4°C were equilibrated at room temperature for 1 hour prior to agitation using a Vial-Mix (Lantheus Medical Imaging) for 45 s, and used at least 15 min after agitation.

### Preparation of human whole blood clots

In accordance with previously reported protocols, cylindrical human clots (initial mean clot width of 302.3±41.8 μm) were formed around silk sutures in micropipettes [22,33,48]. Briefly, borosilicate micropipettes (1.12 mm inner diameter, World Precision Instruments, Sarasota, FL, USA) were cut to a length of 2.5 cm and each was threaded with a 10 cm long 7–0 silk suture (Ethicon Industries, Cornelia, GA, USA). The threaded micropipettes were placed into 10 x 75mm disposable borosilicate glass culture tubes (VWR, West Chester, PA, USA). Following a protocol approved by the University of Cincinnati Institutional Review Board, written consent was obtained from four healthy volunteers and venous human blood was drawn from each volunteer into 10-mL BD plastic syringes (Franklin Lakes, NJ, USA). A 500 μL aliquot of donor blood was pipetted into each of the disposable glass culture tubes containing a threaded micropipette and allowed to clot at 37°C for 3 hrs, followed by a minimum of 3 days at 4°C to allow for retraction [48]. The use of this clot model allowed direct comparison with previously published experiments which investigated mechanisms of thrombolysis in human clots [22].

### Preparation of porcine whole blood clots

The protocol used to prepare human blood clots [22] was combined with a protocol for preparing porcine blood clots [19] to produce porcine whole blood clots around silk sutures. Borosilicate micropipettes (1.12 mm inner diameter, World Precision Instruments, Sarasota, FL, USA) were cut to a length of 2.5 cm, threaded with 10 cm long 7-O silk sutures (Ethicon Industries, Cornelia, GA, USA), and placed into 10x75mm disposable borosilicate glass culture tubes (VWR, West Chester, PA, USA). Porcine clots were created using whole porcine blood anti-coagulated with citrate phosphate dextrose (CPD) solution from Lampire Biological Laboratories, Inc. (Pipersville, PA, USA). The CPD blood was recalcified with 309 mM calcium chloride (Sigma-Aldrich, St. Louis, MO, USA) solution in a 9:1 ratio. Aliquots of 500 μL recalcified blood were pipetted into each glass culture tube. The recalcified blood was allowed to clot for 3 hours in a 37°C water bath and was stored at 4°C for a minimum of 3 days to allow for clot retraction. The initial mean clot width of porcine clots was 384.6±41.8 μm.

### Comparison of rt-PA susceptibility in porcine and human clots using time-lapse microscopy *in vitro*

#### Experimental set-up

**Fig 1:**
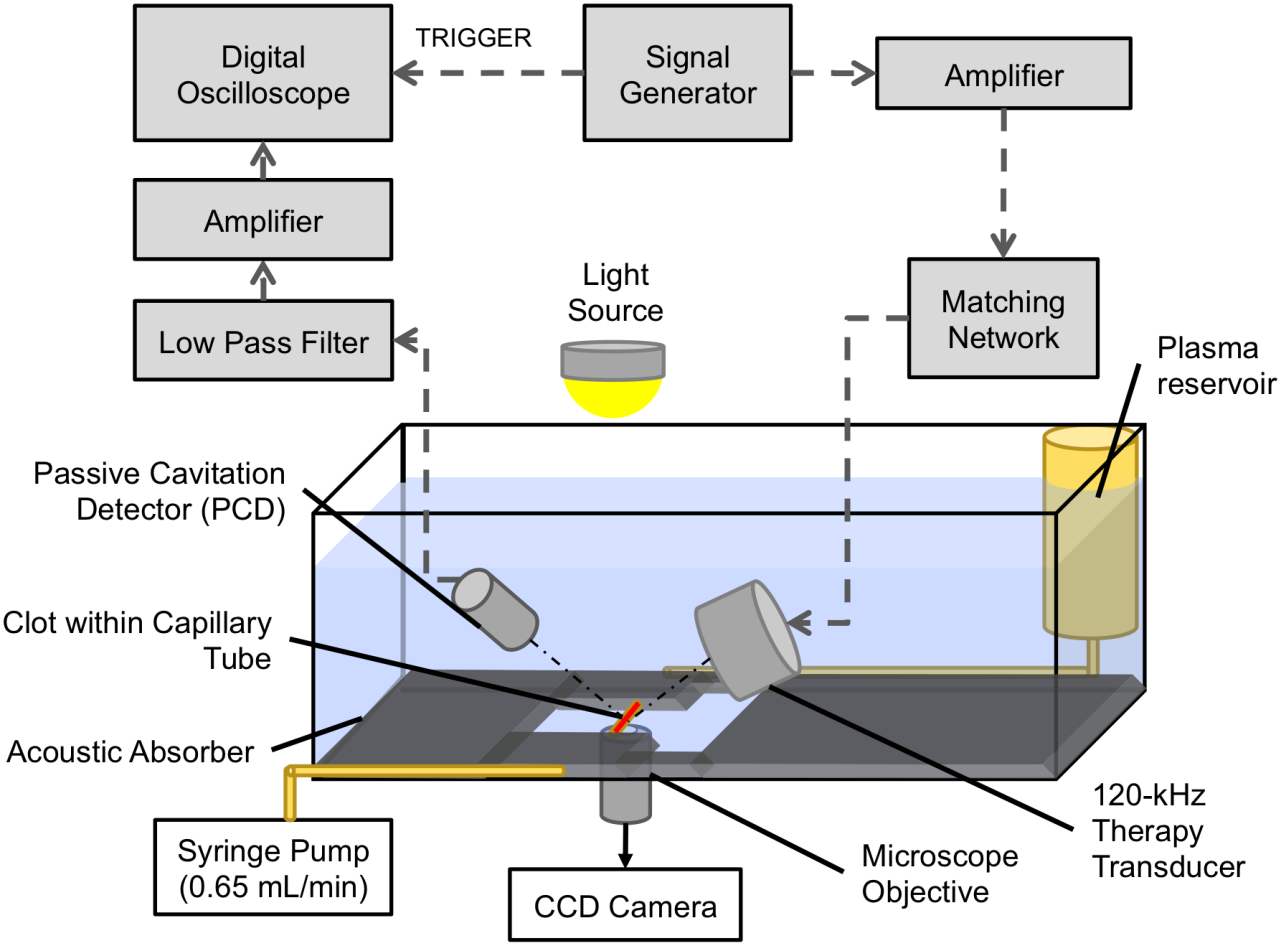
The experimental set-up for the *in vitro* flow system. In a water tank held at 37°C, the clot is mounted within a capillary tube through which plasma is drawn from an upstream plasma reservoir to a downstream syringe pump. Acoustic absorber was used to line the 4 vertical walls of the water tank (not shown) and the bottom of the tank (shown), with approximately 3 cm of the capillary tube visible through a window in the acoustic absorber.

Following previously published protocols [22,23,33], an *in vitro* capillary flow system (Fig 1) was used to measure the thrombolytic efficacy and cavitation activity in human and porcine whole blood clots. An acrylic tank (16 x 33 x 9 cm) lined with 1-cm-thick acoustically absorbent material (Aptflex F48, Precision Acoustics, Dorchester, Dorset, UK) was filled with reverse-osmosis water. The water was heated to 37±1°C, degassed (20±5% dissolved oxygen), and filtered (0.2 μm) via a custom recirculation system for the duration of each experiment. The cylindrical clots adherent to sutures were gently removed from the micropipettes, placed into a larger glass micropipette (2.15-mm inner diameter; Drummond Scientific, Broomall, PA, USA), and attached to the flow channel with latex tubing that allowed the clot to be held securely during the experiments. Low-density polyethylene tubing (inner diameter 1.6 mm; Freelin Wade, McMinville, OR, USA) formed the flow channel connecting the glass micropipette to the perfusate reservoir upstream and the syringe-pump downstream. A syringe-pump (Model 44, Harvard Apparatus, South Natick, MA, USA) was used in continuous withdrawal mode to maintain a flow rate of 0.65 mL/min, which is consistent with physiologic flow rates in ischemic stroke [49]. The clot within the micropipette was positioned 1 mm from the bottom of the tank and imaged with an inverted microscope (IX71, Olympus, Center Valley, PA, USA). Images were recorded with a charge-coupled device (CCD) camera (Regita-2000 R, Q Imaging, Surrey, BC, Canada) at a rate of 2.33 Hz throughout each 30-min experiment.

#### Clot diameter

Using the images recorded with the CCD camera, the clot width as a function of time was determined based on an edge-detection routine published previously [50]. The detected clot width was verified visually following the completion of the routine and adjusted in a few cases to correct algorithmic errors (3.7% of experimental runs). These algorithmic errors arose from issues such as debris flowing in the field of view. The clot diameter in each frame was defined as the average distance between the detected edges over all pixel rows minus the diameter of the suture (58.5±12.0 μm) [22,48]. The fractional clot loss (FCL) and average lytic rate (ALR) were computed to evaluate the thrombolytic efficacy. The fractional clot loss was defined as:

$$\text{FCL}=\frac{{\text{CW}}_{\text{o}}{-\text{ CW}}_{\text{f}}}{{\text{CW}}_{\text{o}}}\times \;100\%,$$ (1)

where CW represents the clot width after the suture has been subtracted. CW_0_ represents the initial clot width, and CW_f_ represents the final clot width after the completion of treatment (30 min).

The average lytic rate was defined as:

$$\text{ALR}=\frac{\text{FCL}}{{\text{t}}_{\text{d}}}\text{,}$$ (2)

where *t*_*d*_ represents the treatment duration. For clots that lysed completely before 30 minutes, *t*_*d*_ was set to the time at which 100% *FCL* was achieved. The ALR was used as a secondary metric to improve assessment of groups in which 100% FCL was achieved prior to the end of the treatment period.

#### Ultrasound insonation and detection

For trials involving US exposure, clots and perfusate were insonated using a custom-designed unfocused 120 kHz transducer (30-mm diameter aperture; H160, Sonic Concepts, Woodburn, WA, USA) [22,23,33]. A function generator (33250A, Agilent Technologies, Santa Clara, CA, USA) was used to generate tone-bursts that were boosted by 55 dB by a power amplifier (1040L, ENI, Rochester, NY, USA). The transducer was excited at 120 kHz, and the power transfer to the transducer was maximized via a custom-built impedance matching network (Sonic Concepts, Woodburn, WA, USA). A peak-to-peak pressure of 0.44 MPa (*in situ*) was employed using an intermittent insonation scheme (50 s continuous wave insonation followed by a 30 s quiescent period) for the duration of the 30-min experiment [20]. The 50-s on-time was found to maximize ultraharmonic (UH) emissions, indicative of stable cavitation [34], throughout the 30-min treatment period [22]. A 30s off-time allowed for replenishment of Definity^®^ within the microscope field of view for the flow rate employed in this study (0.65 mL/min). An intermittent exposure scheme also prevented transducer overheating during the 30-min treatment period.

The acoustic field within the acrylic tank was measured and the transducer was calibrated previously [23]. No standing waves or constructive interference were detected at the location of the clot. A single-element long-focus 2.25-MHz transducer (595516C, Picker Roentgen GmbH, Espelkamp, Germany) was used as a passive cavitation detector (PCD) to measure ultraharmonic and broadband emissions, which are characteristic of stable [20,32] and inertial cavitation [32] respectively. The signal from the PCD was low-pass filtered (10-MHz, J73E, TTE, Los Angeles, CA, USA), boosted by a wide-band low-noise amplifier (CLC100, Cadeka Microcircuits, Loveland, CO, USA), and digitized (10-ms duration, 31.25-MHz sampling frequency). Custom MATLAB scripts were used to analyze the power spectra of the PCD signal. To quantify stable cavitation emissions, the UH energy bands between 250 kHz and 1 MHz were summed over a 2-kHz bandwidth centered around each UH frequency, which were previously shown to have a signal-to-noise ratio greater than 3 dB [23]. To quantify inertial cavitation emissions, the BB energy was summed in 4-kHz bands centered around the UH bands (±10 kHz and ±30 kHz).

#### Experimental protocol

Porcine and human clots were exposed to one of three experimental protocols in the presence of human or porcine plasma, respectively. Each clot was exposed to one of three treatment protocols: plasma alone; plasma with rt-PA (3.15 μg/mL); and plasma with rt-PA (3.15 μg/mL) and Definity^®^ (2 μL/mL) exposed to intermittent 120 kHz US as described above. We also compared the thrombolytic efficacy against previously published studies from our laboratory that exposed human clots to human plasma, rt-PA, Definity^®^, and intermittent 120 kHz US [22]. We employed the same experimental system, utilized the same treatment protocols and analysis scripts, obtained human blood from the same volunteer donor pool, and plasma from the same source as Bader et al (2015). Additionally, porcine clots were also exposed to each of the 3 experimental protocols in porcine plasma for comparison. The rt-PA concentration of 3.15 μg/mL is consistent with steady-state rt-PA concentration in the bloodstream after an intravenous injection of rt-PA in a patient with ischemic stroke [51]. The concentration of Definity^®^ (2 μL/mL, or 2.4 x 10^7^ microspheres/mL [The particle concentration at a 2 μL/mL dilution of Definity^®^ was previously erroneously cited as 1 x 10^4^ particles/mL [22,33].]) was based on our previous studies [22]. In each trial, one clot was mounted in the capillary tube, connected to the flow system, submerged in the water tank, and positioned over the microscope objective. The PCD focus was aligned with the capillary tube and used to record US emissions over the 30 min treatment period. The CCD camera recorded images at a rate of 2.3 Hz, which were transferred to a desktop computer for offline analysis.

### Histology

Human whole blood clots and porcine whole blood clots were fixed in 10% formalin, embedded in paraffin, and stained with hematoxylin and eosin (H&E) before being analyzed histologically. An Olympus IX-71 research inverted microscope and DP72 camera (Olympus Scientific Solutions Americas Inc., Waltham, MA, USA) were used to observe and image the clot sections.

### Electron microscopy

Clots were processed and subjected to routine scanning electron microscopy (SEM) to visualize the fibrin mesh. After the 3-day retraction period, clots were removed from the capillary tubes and rinsed with cold PBS. Clots were placed in 2.0% paraformaldehyde and 2.5% gluteraldehyde in 0.1 M sodium-cacodylate buffer, pH 7.4, for an overnight fixation. Samples were rinsed 3 times with 0.1 M sodium-cacodylate buffer and post-fixed with 1% osmium tetroxide. Samples were again rinsed 3 times with 0.1 M sodium cacodylate buffer and dehydrated in a graded series of ethanol steps for 30-min each (v/v: 25, 50, 75, 95, 100, 100, and 100%). Finally, clots were dehydrated on a Leica EM CPD300 Critical Point Dryer (Leica Microsystems, Buffalo Grove, IL, USA) and sputter-coated with gold-palladium on a Leica EM ACE600 High Vacuum Sputter Coater. High-resolution SEM images (800-7000x magnifications) were acquired from the surfaces of six clot samples (three human and three porcine).

Three blinded observers evaluated the fibrin fiber diameter of the human and porcine clots using ImageJ (National Institutes of Health, Bethesda, MD, USA), based on previously published methods [19]. Each observer was presented with a SEM image (3500x, 8 μm square), on which 5 randomly chosen locations were marked. The diameters of the fibers closest to each location were measured and recorded by the observer.

### Statistical analysis

All data were analyzed in MATLAB (Mathworks, Natick, MA, USA). Lilliefore's test was performed on each data set to assess data for normality. Lytic efficacy, assessed via FCL and ALR, was compared using one-way ANOVA between treatment protocols for each combination of clot and plasma type (porcine clots in porcine plasma, human clots in porcine plasma, and porcine clots in human plasma). Within each treatment (plasma only, rt-PA, or rt-PA and Definity^®^ exposed to US), the lytic efficacy of each combination of clot and plasma type was also compared. The lytic efficacy for human clots in porcine plasma, porcine clots in human plasma, and porcine clots in porcine plasma was directly compared to previously reported lytic efficacy of human clots in human plasma in the same time-lapse microscopy system [22]. ALR was calculated for cross-species experiments to improve analysis of groups that achieved 100% FCL prior to the end of the 30-min treatment period. Post-hoc analysis was done using the Tukey method to identify significant differences in lytic efficacy. Lilliefore's test revealed that the fiber diameter data deviated from normality for both types of clots (p <0.05). Fiber diameter was compared using the Wilcoxon rank sum test.

## Results

### Comparison of rt-PA susceptibility in porcine and human clots

**Fig 2:**
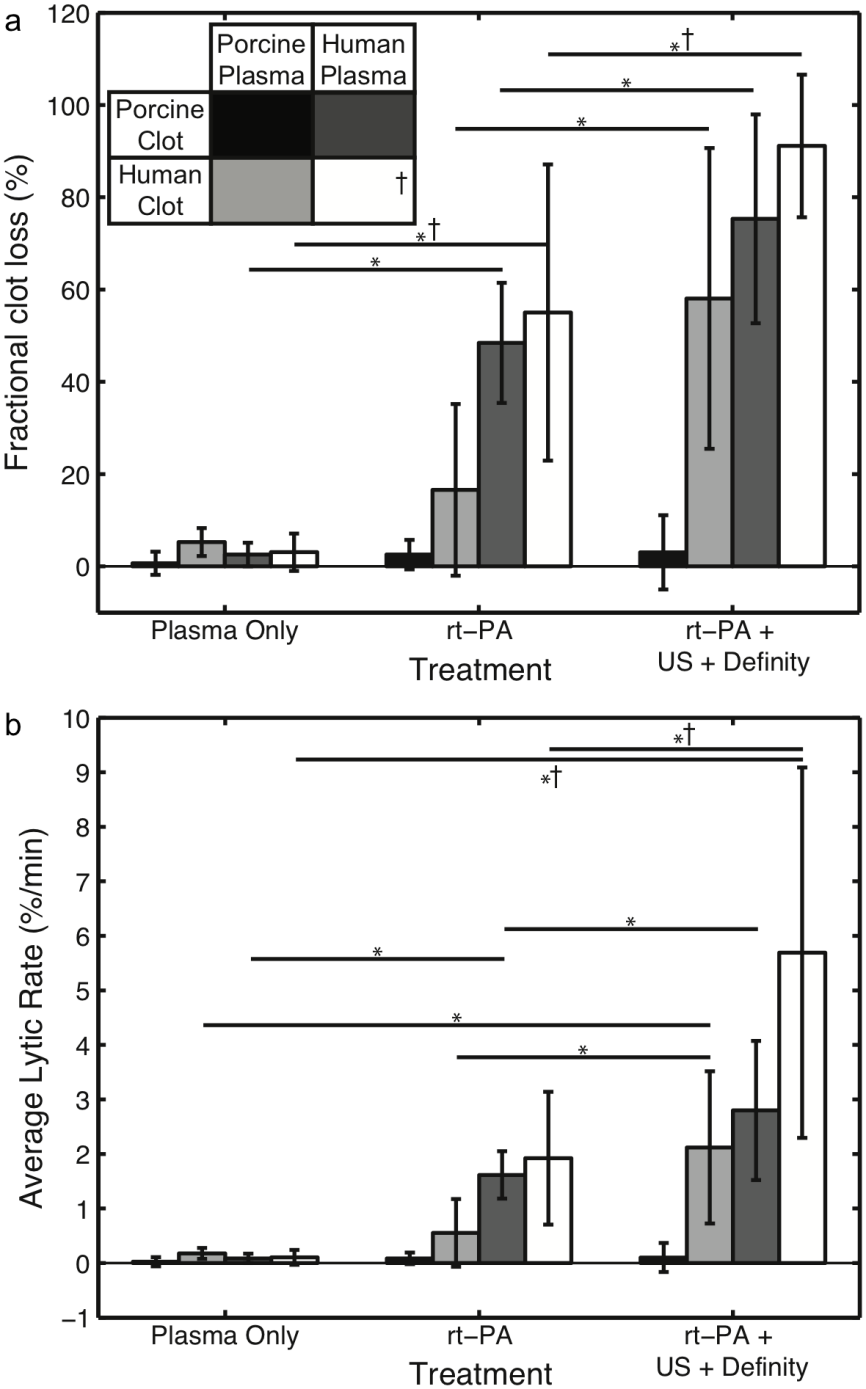
Thrombolysis of human and porcine clots. **(a)** Fractional clot loss (FCL) for porcine clots in porcine plasma (n = 5, black), human clots in porcine plasma (n = 12, light grey), and porcine clots in human plasma (n = 12, dark grey) exposed to plasma alone, rt-PA (3.15 μg/mL), and rt-PA (3.15 μg/mL) with Definity^®^ and intermittent 120 kHz ultrasound (US). Statistically significant differences in FCL (*p*<0.05) across treatments are denoted by (*). No difference (*p*>0.05) in FCL was observed for porcine clots in porcine plasma exposed to rt-PA without or with the use of Definity^®^ and US as an adjuvant compared to plasma alone. **(b)** Average lytic rate (ALR) for the same clots and treatments shown in 2(a). Statistically significant differences in ALR (*p* < 0.05) across treatments are denoted by (*). ^**†**^ Data with human clots in human plasma (n = 12, white) was reproduced from [22].

The fractional clot loss (FCL) at the end of the 30-min treatment period for porcine clots in porcine plasma, porcine clots in human plasma, and human clots in porcine plasma exposed to either plasma alone, rt-PA (3.15 μg/mL), or rt-PA (3.15 μg/mL) and Definity^®^ with intermittent 120 kHz US is shown in Fig 2a. For comparison, previously published data with human clots in human plasma exposed to the same treatment protocols are also plotted [22]. For all clots treated with plasma alone, negligible reduction in clot width was observed (0.67±2.52%, 2.56±2.56%, and 5.27±3.03% for porcine clots in porcine plasma, porcine clots in human plasma, and human clots in porcine plasma, respectively). Porcine clots in porcine plasma treated with rt-PA (3.15 μg/mL) or rt-PA (3.15 μg/mL) and Definity^®^ with intermittent 120 kHz US exposure showed equivalent FCL to clots treated with plasma only (2.56±3.19% and 3.05±8.04% respectively). Additionally, increased thrombolysis was not observed in human clots exposed to porcine plasma treated with rt-PA (3.15 μg/mL) compared to clots in plasma alone (*p*>0.05).

Porcine clots in human plasma treated with either rt-PA (3.15 μg/mL) or rt-PA (3.15 μg/mL) and Definity^®^ with intermittent 120 kHz US exhibited a significantly higher FCL than clots treated with plasma alone (48.44±13.01% and 75.35±22.63%, respectively). Human clots in porcine plasma treated with rt-PA (3.15 μg/mL) and Definity^®^ with intermittent 120 kHz US also exhibited significantly higher FCL than clots treated with plasma alone (58.08±32.61%). For both porcine clots in human plasma and human clots in porcine plasma, treatment with rt-PA (3.15 μg/mL) and Definity^®^ exposed to intermittent 120 kHz US exhibited a significantly higher FCL than treatment with rt-PA (3.15 μg/mL) alone. A full analysis of statistical significance across clot types and treatments may be found in Tables 1 and 2.

**Table 1:** Tukey p-values for cross-species thrombolysis experiments within each combination of clot/plasma.

**A. Porcine Clots, Porcine Plasma**			
	Plasma Only	rt-PA	rt-PA, US, Definity
Plasma Only	--		
rt-PA	0.665	--	
rt-PA, US, Definity	0.527	0.981	--
**B. Human Clots, Porcine Plasma**			
	Plasma Only	rt-PA	rt-PA, US, Definity
Plasma Only	--		
rt-PA	0.432	--	
rt-PA, US, Definity	0.000*	0.000*	--
**C. Porcine Clots, Human Plasma**			
	Plasma Only	rt-PA	rt-PA, US, Definity
Plasma Only	--		
rt-PA	0.000*	--	
rt-PA, US, Definity	0.000*	0.000*	--

* indicates p<0.05.

**Table 2:** Tukey p-values for cross-species thrombolysis experiments within each treatment protocol.

**A. Treatment: Plasma Alone**					
		Porcine Plasma		Human Plasma	
		Porcine clots	Human clots	Porcine clots	Human clots^†^
Porcine Plasma	Porcine clots	—			
	Human clots	0.002*	—		
Human Plasma	Porcine clots	0.394	0.149	—	
	Human clots^†^	0.196	0.308	0.977	—
**B. Treatment: rt-PA (3.15 μg/mL)**					
		Porcine Plasma		Human Plasma	
		Porcine clots	Human clots	Porcine clots	Human clots^†^
Porcine Plasma	Porcine clots	—			
	Human clots	0.637	—		
Human Plasma	Porcine clots	0.002*	0.006*	—	
	Human clots^†^	0.000*	0.000*	0.874	—
**C. Treatment: rt-PA (3.15 μg/mL), Definity (2 μL/mL), & 120 kHz US**					
		Porcine Plasma		Human Plasma	
		Porcine clots	Human clots	Porcine clots	Human clots^†^
Porcine Plasma	Porcine clots	—			
	Human clots	0.000*	—		
Human Plasma	Porcine clots	0.000*	0.278	—	
	Human clots^†^	0.000*	0.007*	0.373	—

* indicates p<0.05.

^†^ Data with human clots in human plasma data previously published [22]

Fig 2b shows the average lytic rate (ALR) for porcine clots in porcine plasma, porcine clots in human plasma, and human clots in porcine plasma, treated with plasma alone, rt-PA, or rt-PA with Definity^®^ exposed to 120 kHz US. Previously published data with human clots in human plasma exposed to the same treatment protocols are also plotted for comparison [22]. The ALR for plasma treated clots irrespective of clot or plasma type was very low, as was the ALR for porcine clots in porcine plasma exposed to any treatment. Increased ALR was not observed in human clots in porcine plasma treated with rt-PA (*p*>0.05), but was observed in porcine clots in human plasma treated with rt-PA (*p*<0.01). An increased ALR was observed for both combinations treated with rt-PA and Definity^®^ exposed to 120 kHz US (*p*<0.01).

**Fig 3:**
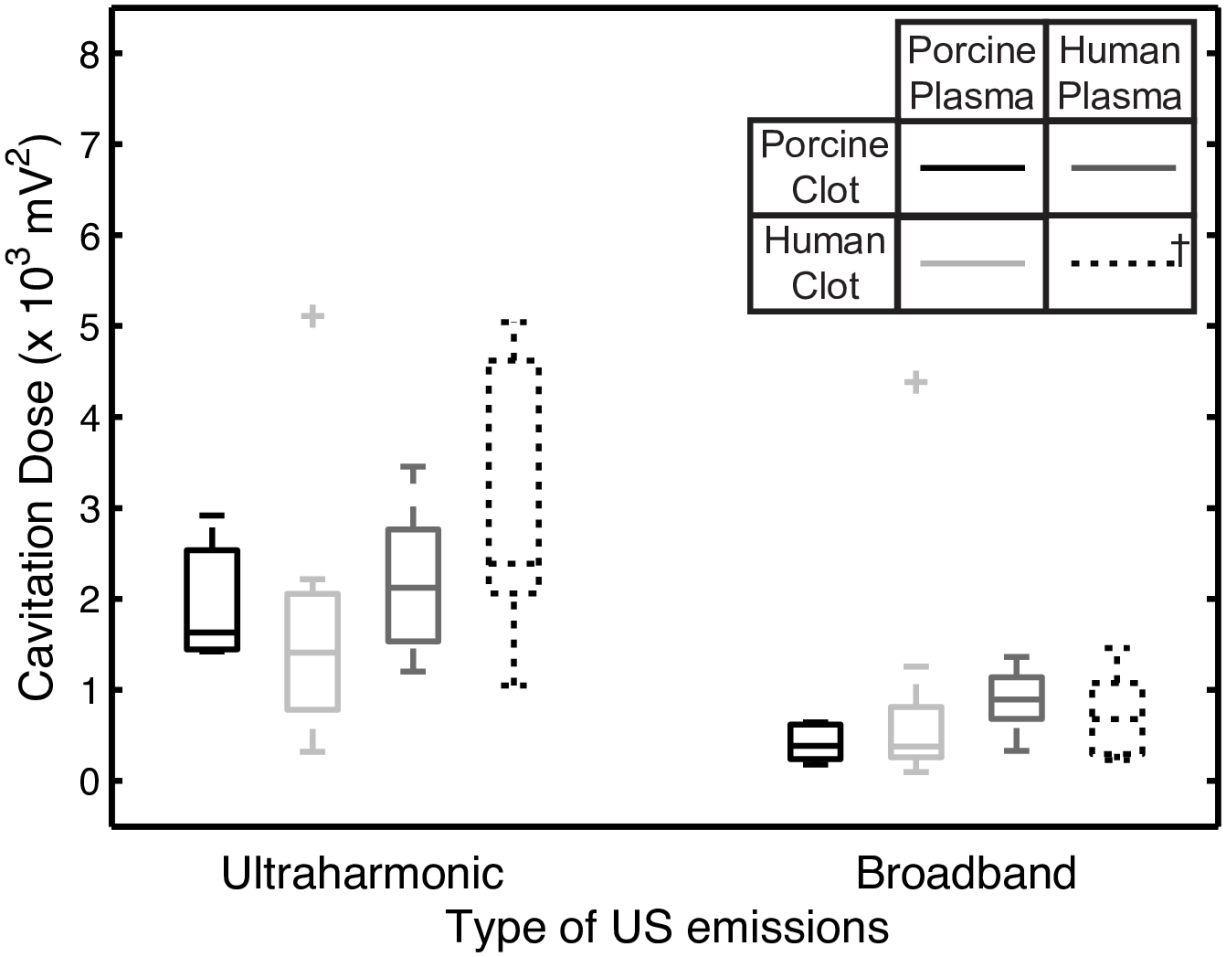
Measured ultraharmonic and broadband emissions for trials involving US exposure. Cavitation dose is shown for porcine clots in porcine plasma (black), human clots in porcine plasma (light grey), and porcine clots in human plasma (dark grey) when treated with rt-PA, Definity^®^, and 120 kHz US. ^**†**^ Data with human clots in human plasma (dashed line) was reproduced from [22].

The ultraharmonic (UH) and broadband (BB) energy detected by the PCD during trials with US exposure are shown in Fig 3. Again, previously published data with human clots in human plasma are plotted for comparison [22]. The amount of UH energy detected is not significantly different (*p*>0.05) for porcine clots in porcine plasma, porcine clots in human plasma, or human clots in porcine plasma.

### Histological examination of the clots

**Fig 4:**
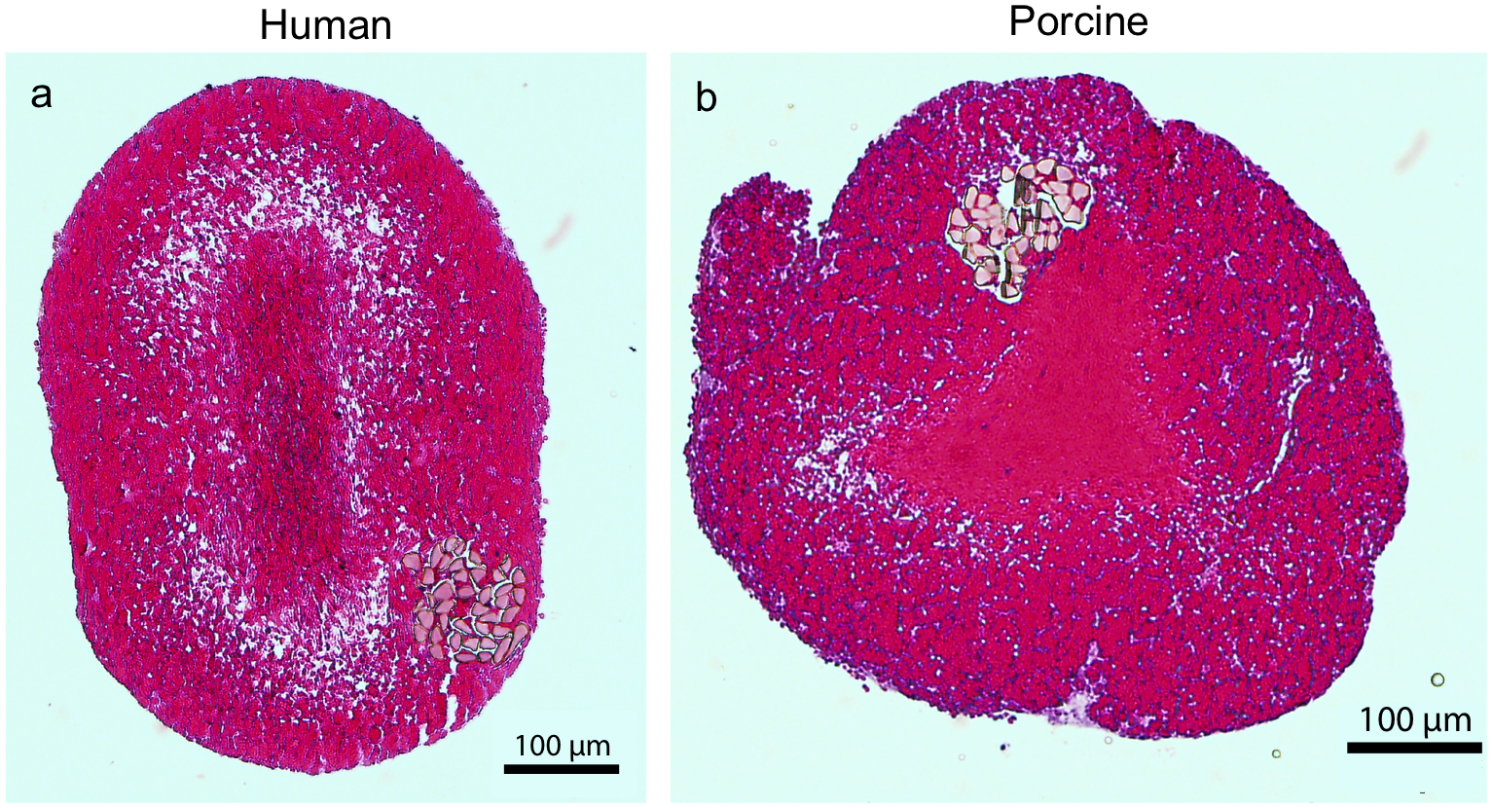
Representative human (a) and porcine (b) clots with standard H&E staining.

Histological examination of the human whole blood clots (Fig 4a) sectioned axially revealed erythrocyte-rich clots with a dense central region and a porous outer layer. Examination of the porcine blood clots (Fig 4b) revealed a similar appearance as the human blood clots: erythrocyte-rich with a similar degree of porosity near the surface of the clots. Porcine clots also had a central region of increased clot density that was surrounded by a region of increased porosity.

### SEM examination of the clots

**Fig 5:**
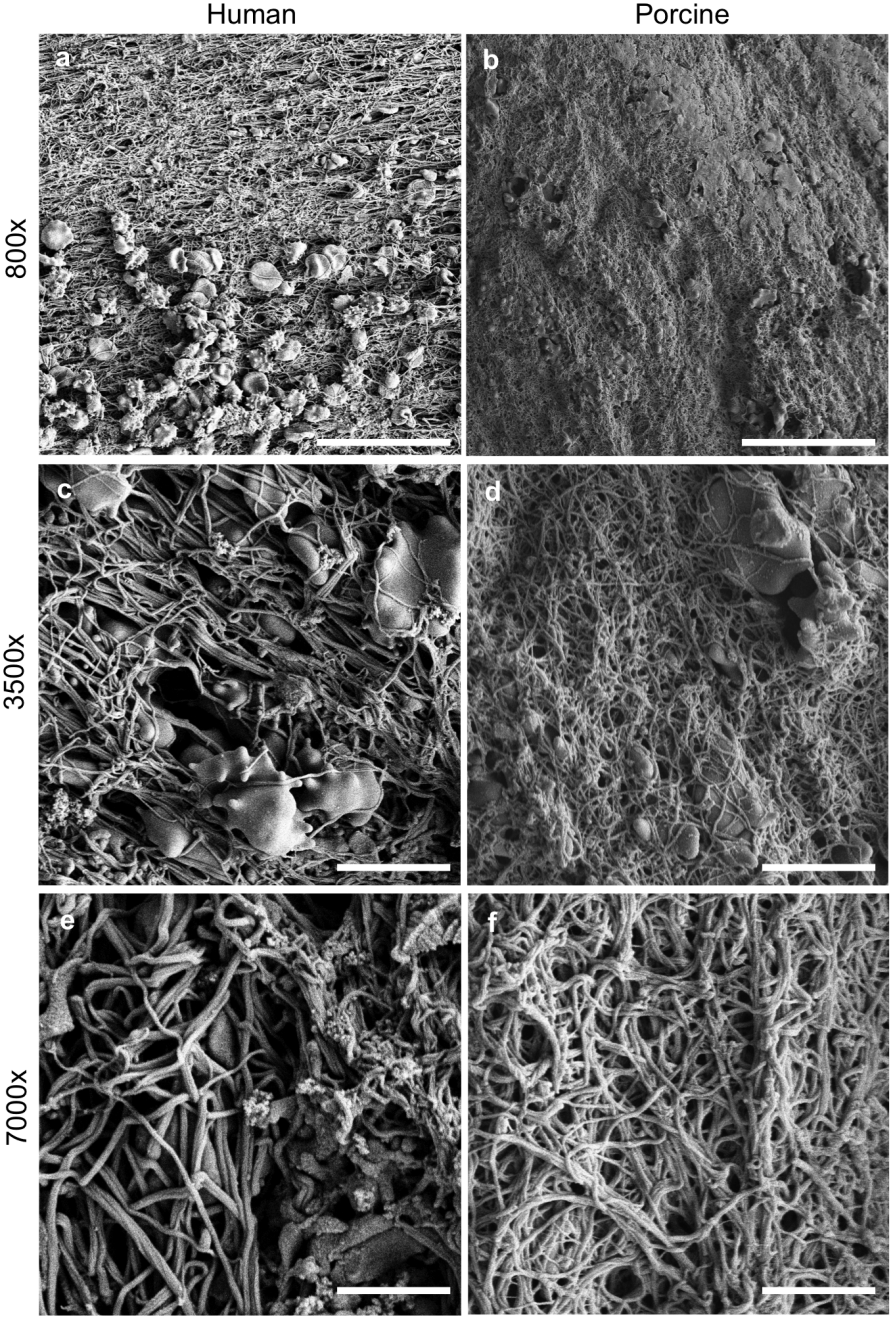
Representative human (a, c, e) and porcine (b, d, f) SEM images of clot surfaces. Images are at 800x magnification (**a, b**; bar = 25 μm), 3500x magnification (**c, d**; bar = 5 μm), and 7000x magnification (**e, f**; bar = 2.5 μm)

**Fig 6:**
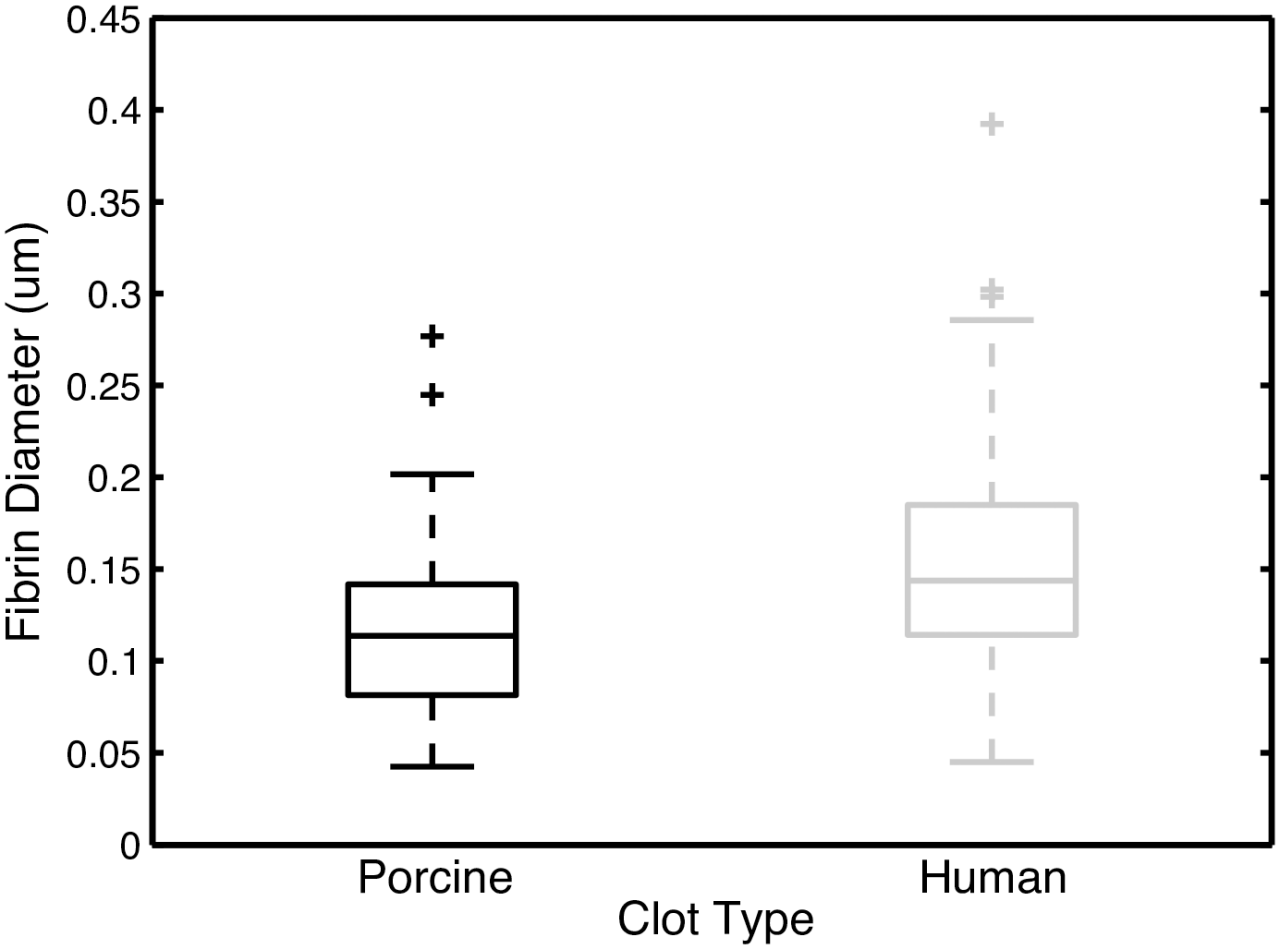
Clot fibrin fiber diameter. Box plots showing the range, median, 25th quartile, and 75th quartile of measured fiber diameter for human (n = 130) and porcine (n = 140) clots as measured by blinded observers from SEM images. Outliers are represented by the symbol "+" and statistically significant differences are denoted with a "*".

Scanning electron microscopy revealed both qualitative and quantitative differences in clot structure and composition between human and porcine clots. Representative SEM images of the surface of each clot are shown in Fig 5. Porcine clots had fewer visible erythrocytes on the surface of clots compared to human clots (Fig 5a and 5b) and exhibit a denser fibrin mesh composed of thinner fibrin fibers compared to human clots (Figs 5 and 6). Porcine clots had a median fibrin fiber diameter of 0.114 μm, which is significantly different from the median human fiber diameter of 0.144 μm (*p*<0.01) (Fig 6).

## Discussion

In this study we evaluated thrombolysis in human and porcine clots in the presence of flow treated with rt-PA and Definity^®^ exposed to intermittent 120 kHz US. The flow rate used in this study is within the range observed in the middle cerebral artery during ischemic stroke [49]. The presence of flow is known to impact both cavitation activity and duration as well as thrombolytic activity. Specifically, flow allows the constant replenishing of Definity^®^ as well as the removal of fibrin degradation products, which makes the drug delivery setting different compared to experiments without flow [52]. Further, the peak-to-peak pressure used in this study was low (0.44 MPa) and sustained stable cavitation activity was observed (Figs 3 and 5). Shimizu *et al*. investigated the effect of 490-kHz continuous wave US (peak-to-peak pressure of 0.25 MPa) on monkey brains and did not observe tissue damage or neurological deficits [53]. A 2005 clinical trial employing 300-kHz continuous wave US at an intended peak to peak pressure of 0.12 to 0.26 MPa noted an increased rate of sICH [54]. A post-hoc analysis revealed that the increased sICH rate was likely caused by the presence of US standing waves within the cranium with *in situ* peak to peak pressures between 0.54 and 2.4 MPa [55]. Clearly careful selection of US exposure parameters is required to promote enhanced thrombolysis and mitigate adverse bioeffects.

The types of thrombi that cause ischemic stroke *in vivo* are highly variable in both their composition and susceptibility to rt-PA lysis [38,41,56]. Hashimoto *et al*. found that of 83 stroke patients with thrombectomy, 41% had erythrocyte-rich thrombi [42]. Liebeskind *et al*. found that 56% of clots retrieved from 50 stroke patients were either predominantly erythrocytic or had a mixture of fibrin and erythrocytes [57]. Marder *et al*. (2006) provided a qualitative description of clots removed from 25 stroke patients, of which 12% were predominantly erythrocytic, 12% were fibrin-rich, and 76% were a mixture of fibrin and erythrocytes.

Our experiments showed that porcine clots exposed to porcine plasma treated with rt-PA (3.15 μg/mL), without or with Definity^®^ and US, exhibited negligible thrombolysis. However, porcine clots exposed to human plasma treated with rt-PA (3.15 μg/mL) exhibited a similar FCL as human clots in human plasma exposed to rt-PA at the same concentration. Furthermore, the addition of Definity^®^ and 120 kHz US increased FCL for both porcine clots in human plasma and human clots in porcine plasma. US-enhanced thrombolysis is thus observed for human clots in human plasma, but not for porcine clots in porcine plasma (Fig 2).

Our results shown in Fig 2a are consistent with the work of Flight *et al*., which revealed a higher activation of human plasminogen in response to rt-PA than porcine plasminogen [46]. If porcine clots are exposed to porcine plasma, only porcine plasminogen is present, which produces a substantially lower concentration of active plasmin, leading to lower thrombolytic efficacy relative to human clots in human plasma exposed to the same concentration of rt-PA. The reduced rt-PA thrombolysis for human clots in porcine plasma appears to be caused by the lack of human plasminogen in the surrounding fluid, despite the presence of human plasminogen intercalated within the clots. The presence of human plasminogen in the surrounding fluid enables rt-PA thrombolytic efficacy, regardless of the type of clot (human or porcine).

The adjuvant use of Definity^®^ and 120 kHz US improved rt-PA thrombolytic efficacy for human clots in porcine plasma over rt-PA alone. Previous studies have concluded that the addition of Definity^®^ and 120 kHz US exposure enhances the penetration of thrombolytic into the clot [20,32]. Acoustically activated Definity^®^ microbubbles likely serve as micropumps which increase the transport of rt-PA further into the clot allowing activation of the human plasminogen within the clot. The UH dose measured during exposure to US (Fig 3) was comparable to that reported previously by Bader *et al*. [33]. We observed a similar degree of US enhancement of rt-PA thrombolysis (increase in FCL of 41.5%) for human clots in porcine plasma as Bader *et al*. observed for human clots in human plasma (increase in FCL of 36.1%) (Fig 2a).

Equivalent rt-PA lytic efficacy was measured for porcine clots in human plasma and human clots in porcine plasma exposed to rt-PA with Definity^®^ and US (Fig 2a) despite the large difference in the amount of human plasminogen present in the system. The amount of human plasminogen in the human clots given their volume is estimated to have on the order of 0.002–0.004 U of activity, which is 4 orders of magnitude smaller than amount of human plasminogen in the human plasma flowing by either type of clot (18–28 U). The co-localization of plasminogen and rt-PA at the site of fibrin is likely key in producing rt-PA thrombolysis. Definity^®^ and US exposure improves the penetration of both proteins. Site-specific delivery of plasmin to the clot is an alternative strategy to reduce the dependence on the *in situ* availability of plasminogen [58]. Our group has reported thrombolysis using plasmin that was encapsulated into echogenic liposomes to prevent inhibition in the bloodstream [58].

In studies by Landskroner *et al*. (2005), 5 mg human plasmin produced 50±4% mass loss from porcine clots, compared to 80±2% mass loss from human clots after a 60-min treatment period [44]. Landskroner *et al*. attributed this decreased thrombolysis in porcine clots to a denser fibrin network as observed on scanning electron microscopy [44]. Our study also showed that porcine clots have a denser fibrin network (Fig 5) with smaller diameter fibrin diameters than human clots (Fig 6). In addition, the ALR for porcine clots in human plasma treated with rt-PA with Definity and US was also significantly lower than the ALR for human clots in human plasma (Fig 2b, *p*<0.01). A denser fibrin network, characterized by decreased strand thickness and increased fibrin density, has been correlated with decreased rt-PA susceptibility in human thrombi *ex vivo* and clots *in vitro* [45,59]. The denser fibrin network in porcine clots may be attributable to the increased levels of coagulation factors in swine blood (Table 3) [60]. Platelets are known to inhibit fibrinolysis [61]. Therefore, the higher number of platelets in porcine blood [60] could also have contributed to the lower rt-PA thrombolytic efficacy in porcine blood clots observed in this study.

**Table 3:** Human and porcine blood composition and coagulation characteristics.

Coagulation parameter	Human (normal range)	Porcine (n = 6)
Factor I (mg/dL)	150–450	348 ± 78
Factor II (U/mL)	0.70–1.30	0.68 ± 0.3
Factor V (U/mL)	0.65–1.45	2.80 ± 0.6
Factor VII (U/mL)	0.50–1.30	1.92 ± 0.4
Factor VIII (U/mL)	0.75–1.40	6.42 ± 0.8
Factor IX (U/mL)	0.65–1.0	3.20 ± 0.3
Factor X (U/mL)	0.75–1.25	2.54 ± 0.9
Factor XII (U/mL)	0.50–1.45	5.65 ± 1.4
Plasminogen (U/mL)	0.80–1.20	0.50 ± 0.1 (n = 2)
Platelet count (x 10^3^/mm^3^)	150–450	580 ± 115 (n = 9)
Clotting Time (min)	6–12	6 ± 3.8
Hematocrit (%)	37–52	38 ± 2.1

Values were compiled from Lewis' *Comparative Hemostasis in Vertebrates* [60]

The effect of clot structure on rt-PA thrombolytic susceptibility as also been previously investigated by our laboratory. Sutton *et al*. found that the structure of porcine whole blood clots is affected by the type of glass used to initiate clotting [19]. Specifically, unretracted clots manufactured in flint glass have lower fibrin content and are less dense and more porous than the highly retracted clots formed in borosilicate glass. The higher charge or hydrophilicity of borosilicate glass may contribute to a higher degree of platelet activation and increased clot retraction. Unretracted clots exhibited significantly more mass loss than retracted clots, and rt-PA thrombolysis was enhanced with the addition of Definity^®^ and intermittent 120 kHz US [19].

This study possesses a few notable limitations. Our experiments were completed in an *in vitro* flow system, which limits direct applicability to either *in vivo* animal studies or human trials. For example, the *in vitro* flow model used in this study lacked physiological pulsatile blood pressures, and the flow rate was fixed for the entire treatment period [22]. However, the *in vitro* experiments reported in our paper allowed direct quantitation of the differential rt-PA efficacy and the combination of blood products from different species.

The human and porcine whole blood clots used in this study were formed using equivalent protocols, but the human clots were produced using fresh blood, and the porcine clots were produced using recalcified anticoagulated blood. There is evidence that a high concentration of citrate reduces the levels of Factor V, VIII, and IX in human fresh frozen plasma [62]. However, the 3 hr incubation of our clots at 37°C to allow clotting should provide sufficient time for the activation of the coagulation cascade, as the activated partial thromboplastin time in pigs is <30 s [60].

The human clots produced from venous blood donated from healthy volunteers may not completely model thrombi occurring in stroke [63,64], which can vary in composition and morphology [65]. Our human clot model, though erythrocytic, is allowed to retract for 3 days, which leads to a decrease in rt-PA thrombolytic susceptibility [63] and is consistent with a tighter fibrin network [59]. Porcine clots are further resistant to rt-PA thrombolysis and the dilution of whole blood with an anticoagulant and calcium chloride decreases effective hematocrit, noticeable as a decrease in surface erythrocytes on SEM (Fig 5). Additionally, we did not investigate any possible immune reactions that might arise from the combination of blood products from two different species (human and porcine). However, porcine plasmin has formerly been administered to human patients repeatedly without allergic problems [66], demonstrating significant homology between the proteins. Additionally, histology and SEM imaging was performed only on untreated clots.

Although retracted porcine thrombi have been used to model ischemic stroke [36,37,67], porcine studies that have evaluated the efficacy of microbubbles and ultrasound have thus far only used fresh thrombi [24,35]. Retracted porcine thrombi may represent a “worst case” scenario for rt-PA treatment and could serve as an excellent model for certain subtypes of thrombi responsible for ischemic stroke, chronic deep vein thrombosis, and pulmonary emboli. In order to mimic the rt-PA efficacy of human clots, porcine clots may be altered biochemically to model the thrombolytic susceptibility of human clots more closely. Human clots exposed to porcine plasma and rt-PA demonstrated increased lytic susceptibility over porcine clots in porcine plasma (Fig 2a), likely because of co-localization of human plasminogen at the site of lytic activity. In this scenario, the only source of human plasminogen was intercalated in the clot. One approach to "humanize" porcine clots to model human thrombus rt-PA susceptibility may be to dope the porcine whole blood with human plasminogen during the manufacturing process. Alternatively, platelet activation could be modulated to reduce the retraction of clots [68], either chemically or by changing the surface properties of container used to form clots [19].

## Conclusions

The results of this study demonstrate that treating porcine clots in porcine plasma with rt-PA *in vitro* produces 22-fold lower lytic efficacy than treating human clots in human plasma with rt-PA. Including human plasminogen, either at the time of clot formation or in the surrounding plasma, produces more humanoid thrombolysis. Porcine clots in human plasma showed equivalent fractional clot loss for all treatment protocols compared to human clots in human plasma. Human clots in porcine plasma did not demonstrate rt-PA lysis, but the addition of Definity^®^ and 120 kHz US exposure significantly increased lytic efficacy. The use of porcine clot models to test new human thrombolytic therapies may necessitate modulation of coagulation and thrombolytic factors to reflect human hemostasis accurately.
